# Lack of awareness among surgeons regarding safe use of electrosurgery. A cross sectional survey of surgeons in Pakistan

**DOI:** 10.1016/j.amsu.2019.11.017

**Published:** 2019-11-30

**Authors:** Awais Amjad Malik, Romaisa Shamim Khan, Ruqayya Naheed Khan, Osama Shakeel, Hashim Hussnain Ahmed, Uzair Rahid, Anam Fatima, Muhammad Farooq Afzal, Shahid Khattak, Amir Ali Syed

**Affiliations:** a Lahore General Hospital, Pakistan; b Shaukat Khanum Memorial Cancer Hospital, Pakistan

**Keywords:** Electro surgery, Electrocautery, FUSE, Surgical energy, Pakistan

## Abstract

**Objective:**

To assess our surgeons perceptive regarding the safe usage of electrosurgical devices.

**Method:**

ology: This cross sectional survey was carried out at two hospitals, A cancer hospital and a public sector general hospital. Consultants, fellows and senior residents (Resident year 3rd and year 4th) on the surgical floor were requested to fill up the questionnaire. Calculations were performed with Statistical Package for the Social Sciences (SPSS 20) for Windows version 20 statistical software. Data was described using median with minimum and maximum value for quantitative variables. For categorical variables, number of observations and percentages were reported. The study is complied with hospital guidelines on research involving human subjects.

**Results:**

Out of 80 questionnaires 52 were filled and returned. 12 consultants, 16 fellows/Senior registrars and 24 senior residents filled their questionnaires. For the sake of anonymity no information was obtained regarding the level of training and experience. Total 12 questions were asked. An expert level was set for a score above 10/12. A moderate level was set at 8/12. A score of less than 8 was considered unsafe for using electrosurgical devices. Only 6 (11.5%) participants had an expert level of understanding. 16 (30.7%) had moderate understanding. 30 (57.7%) were considered unsafe regarding use of electrosurgical devices. 85% participants were not aware of the correct mode of current to use for coagulating vessels. 69% of surgeons would use electrocautery to control staple line bleeds. 67% participants weren't aware of the correct placement of dispersive electrode. 60% couldn't identify a safe device for use in patients with a pacemaker. 46% of surgeons would cut a dispersive electrode to fit it on a child. 69% believed that harmonic scalpel was a bipolar cautery. 61% couldn't differentiate between RFA and Microwave Ablation. 63% didn't know how to handle an operating room fire.

**Conclusion:**

In these two hospitals, high level of ignorance noticed regarding the procedure and indications of basic electrosurgical equipment which needs raising awareness and further training.

## Highlights

• The first study to show the lack of understanding of electrosurgical devices among surgeons in Pakistan.
• Study was performed in 2 hospitals and included consultants, fellows and residents
• A survey was distributed and questions pertinent to the use of electrosurgical devices were asked.
• Surgical residents, fellows and consultants were equally unaware of how these devices work.
• Study highlights the need to start training courses for these devices.

## 1 Introduction

Ever since the first description of cautery by Bovie in earlier twentieth century, there has been tremendous development in the use of electrosurgical devices. All surgical procedures require some form of electrosurgical device to be performed safely [1].Newer products have been developed, which are very complex and are very task specific. Cavitron ultrasonic surgical aspirator (CUSA), Ligasure and harmonic Ace are a few common examples. Regardless of the specialty, the development of these energy devices have revolutionized the field of surgery [2].

Unfortunately the surgical curriculum is not designed to teach surgeons the safe and effective way of handling these devices [3]. Electrosurgical devices can lead to severe complications such as operating theater fires, burns at the returning electrode site, accidental tissue injury and interference with other implantable medical devices (e.g., pacemakers, implantable cardiac defibrillators [5,6]. One important factor is that the devices tend to be poorly understood by operators, regardless of their level of experience [3].

There are many reports of iatrogenic injuries related to energy devices [[7], [8], [9], [10], [11], [12]]. In the US alone, 600 cases of operating room (OR) fires are reported annually, which can lead to devastating morbidities and mortalities [5]. The prevalence of bowel injuries due to electro surgery during laparoscopic surgery is estimated at 1–2 per 1000 patients [7], with high morbidity related to unrecognized injuries [[7], [8], [9], [10]]. With over 2 million laparoscopic procedures done annually in the United States, this represents a significant safety issue impacting thousands of patients every year. These complications have become one of a major cause of litigations in US [13].

Society of American gastrointestinal and endoscopic surgeons (SAGES) recently assessed the baseline knowledge of 48 of its leaders using an 11-item multiple-choice question examination, testing their knowledge of the principles and adverse events related to the use of energy devices [4]. The median number of correct answers was 59% (IQR 55–73%). Thirty-one percent of SAGES leaders did not know how to correctly handle an operating room fire on the patient; 31% could not identify the device least likely to interfere with a pacemaker; 13% did not know that thermal injury can extend beyond the jaws of a bipolar instrument; and 10% thought that a dispersive pad should be cut to fit a child. In an effort to address this knowledge gap and safety issue, SAGES has developed the fundamental use of surgical energy (FUSE) program. FUSE is the first comprehensive educational tool consisting of a curriculum in the basic science of surgical energy, developed by a multidisciplinary team of content experts (including surgeons, anesthesiologists, nurses, and engineers) [4].

Little evidence has currently been generated to see the understanding of local surgeons regarding safe use of electro surgery in Pakistan. In Pakistan, although the reporting of adverse events is uncommon and in fact discouraged [16,17] we still have our share of the problem. Saaiq et al. reported a series of 3 cases with cautery burn due to misplaced dispersive electrode [14]. A study regarding usage of electrocautery among surgical residents in Karachi was done and showed that there is a huge knowledge gap between the understandings of residents on this topic [15]. Surgical curriculum in Pakistan is no different from the rest of the world and our surgical textbooks hardly cover any aspect on the safe use of electro surgery.

Before we can say, there is a need to start training our surgeons in using devices; we need to see where our surgeons stand regarding understanding and the safe usage of these devices. We therefore designed a cross sectional survey containing questions pertinent to safety of electrosurgical devices.

## 2 Methodology

The study was a cross sectional survey carried out at two hospitals. A specially designed questionnaire was circulated and filled up anonymously by all the consultants, fellows and senior residents (Resident year 3rd and year 4th) on the surgical floor. Anonymity of name and level of training was kept to get maximum participation and also to ascertain the fact that the knowledge regarding safe use of electrosurgical devices is mandatory and basic and is supposed to be acquired across all levels of training. The work has been reported in line with the STROCSS criteria [20].

The questionnaire was distributed among the surgical fraternity of both the hospitals in June 2017 and Oct 2017 respectively. Forms were handed out during the surgical meeting in each hospital. For the sake of anonymity no information was obtained regarding the level of training and experience on the questionnaire. Total 12 questions were asked. An expert level was set for a score of 10/12. A moderate level was set at 8/12. A score of less than 8 was considered unsafe for using electrosurgical devices.

Calculations were performed with Statistical Package for the Social Sciences (SPSS 20) for Windows version 20 statistical software. Data was described using median with minimum and maximum value for skewly distributed quantitative variables. For categorical variables, number of observations and percentages were reported. The study is complied with the hospital guidelines on research involving human subjects.

## 3 Results

**Fig. 1:**
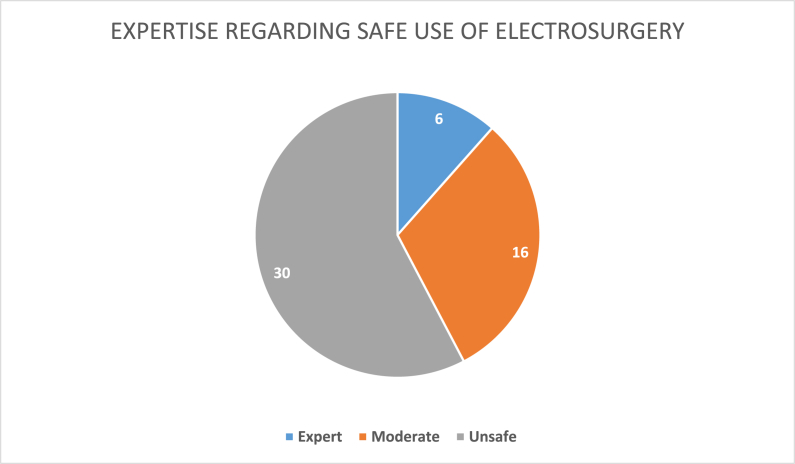
Expertise regarding safe use of electrosurgery.

**Fig. 2:**
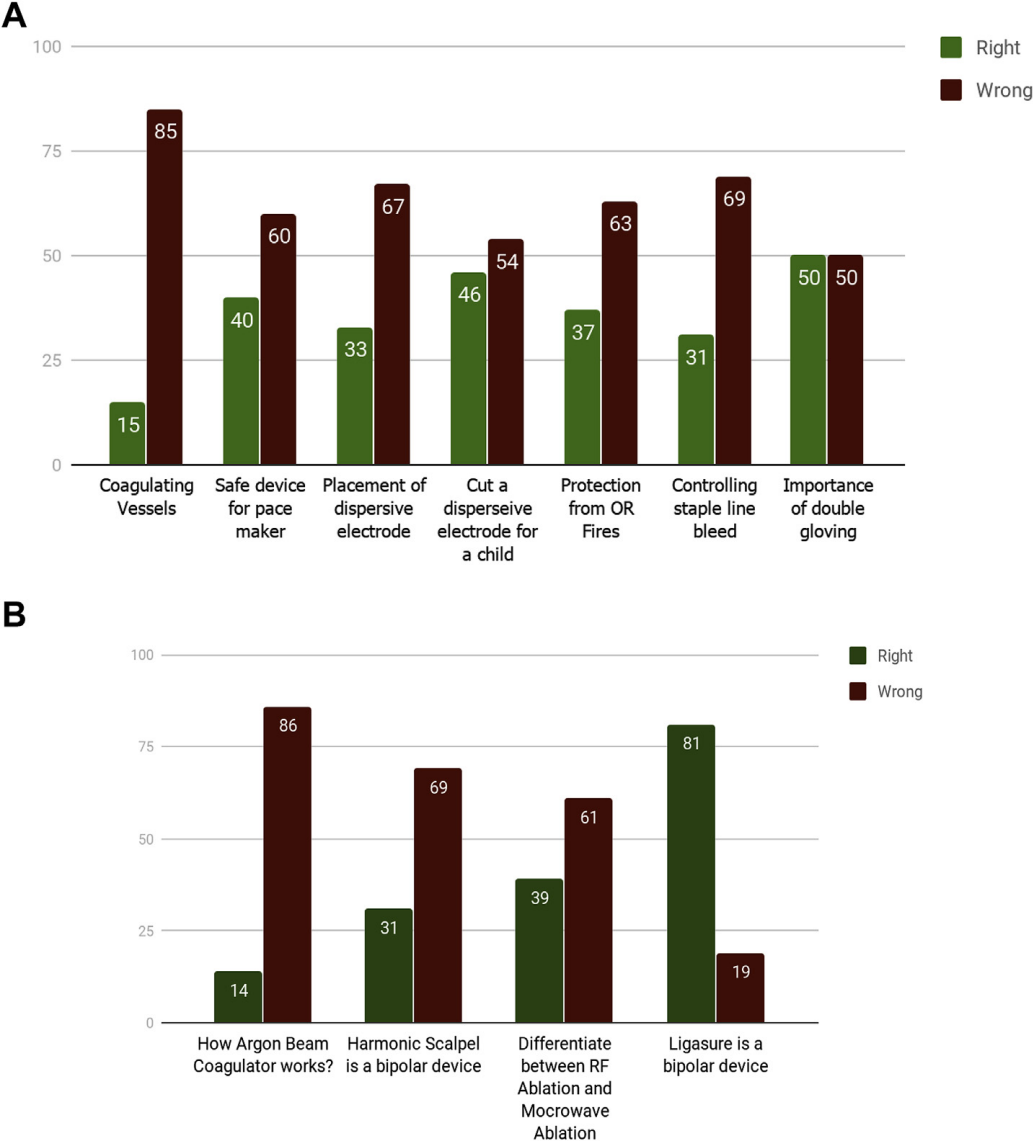
**a**. Response to questions regarding safe use of electrocautery. **b**. Response to questions regarding safe use of electrocautery.

A total of 52 questionnaires were filled and returned. 12 consultants, 16 fellows/Senior Registrars and 24 senior residents were present in the meetings and filled their questionnaires. Only 6 (11.5%) participants had an expert level of understanding. 16 (30.7%) had moderate understanding. 30 (57.7%) were considered unsafe regarding use of electrosurgical devices (Fig. 1). 85% participants were not aware of the correct mode of current to use for coagulating vessels. 69% of surgeons would use electrocautery to control staple line bleeds. 67% participants weren't aware of the correct placement of dispersive electrode. 60% couldn't identify a safe device for use in patients with a pacemaker. 50% participants were unaware of the need for double gloving while using electrocautery. 46% of surgeons would cut a dispersive electrode to fit it on a child. 86% didn't know how the argon beam coagulator worked. 69% believed that harmonic scalpel was a bipolar cautery. 61% couldn't differentiate between RFA and Microwave Ablation. 63% didn't know how to handle an operating room fire. 50% weren't aware how to protect themselves from the hazardous OR smoke. Only 19% were unaware that ligasure is a bipolar cautery (Fig. 2a & b). A comparative assessment between the surgical expertise and level of understanding couldn't be established because this information was not recorded in the questionnaire.

## 4 Discussion

There is lack of understanding on how electrocautery works and how to use it safely. Last few years have seen a multitude of researches pointing out the deficiencies of surgeons regarding the use of electrosurgical device [3,4]. Unfortunately the surgeons in our country are no better [19]. Our study has shown that there is a huge knowledge gap of surgeons on how these things work. The fact that we use these devices on daily basis without actually knowing how these works is disturbing.

Our surgeons are truly lacking in knowledge and understanding of electrocautery and other energy devices. 85% of the surgeons weren't aware of the correct form of current to use for coagulating vessels. 60% of our surgeons couldn't decide which device was safe for use in a patient with a pacemaker. 67% lacked knowledge of how to fit a dispersive electrode, and 54% thought it's safe to cut a dispersive electrode to fit it on a child. All these results are worse than what was seen in the study done by SAGES [4].

Not only are the operating surgeon making the surgery unsafe for the patient but they are also making it unsafe for themselves. Only 50% of the surgeons knew the importance of double gloving. 63% were unaware what to do when a fire breaks out in OR. 50% surgeons didn't know how to protect themselves from the smoke generated by electrocautery.

Things were worse when we moved to newer energy devices such as Harmonic and Argon Beam coagulator. 86% were unaware of what is an argon beam coagulator. 69% thought harmonic was a bipolar cautery device. 61% couldn't differentiate between RF ablation and Microwave ablation. Although these devices aren't commonly used but the knowledge and understanding of how these things work is of utmost importance for the practicing surgeon.

The incidence of operating room fires and other injuries attributable to the energy devices is on the rise [5,7,8]. Even in Pakistan we have a few reports of such incidents [14]. There is a need to arrange hands on workshops and lectures to increase awareness on the safe use of electrosurgical devices which are a necessary part of a surgeons practice.

In an effort to address this knowledge gap and safety issue, SAGES has developed the FUSE program [18,19]. FUSE is the first comprehensive educational tool consisting of a curriculum in the basic science of surgical energy, developed by a multidisciplinary team of content experts (including surgeons, anesthesiologists, nurses, and engineers). It consists of a web-based multimedia curriculum (also available in book format) and a validated certification examination. However, there is no hands-on training or assessment component. The curriculum covers ten domains and addresses 63 objectives and emphasizes safety for all members of the operating room, while keeping the content clinically relevant with practical information, regardless of subspecialty [18].

A lot of evidence now exists reporting the effectiveness of FUSE program. Till date more than 400 surgeons have been trained and are benefitting from the program. The program has now extended to outside the USA and further centers are being validated [19]. Recently the Federation of Visceral and Digestive Surgery (FCVD) in France has taken up the SAGES FUSE course and the surgeons have shown increased satisfaction with the program [21].

We were unable to show a relationship between level of training and level of safety in usage of these energy devices. We believe that the knowledge of energy devices is an essential part of surgical training and everyone irrespective of their level of training should be up-to-date in this. Ally Ha et al. had shown that Surgeons and surgical trainees both have a significant knowledge gap in the safe and effective use of surgical energy devices, regardless of surgical specialty and despite what they feel was adequate training. The knowledge gap is not improved with experience [22].

Surgical training in Pakistan lacks specific training on how these devices work and what is the appropriate way to use them. Considering its importance both for surgeons and patient safety, there is a need to inculcate this training into the core curriculum of all surgical specialties. Workshops need to be organized to train the already practicing surgeons. FUSE course is one such forum which can help in this regard. The college of physicians and surgeons, Pakistan can take up the FUSE course and make it mandatory for all surgical specialties.

## 5 Conclusion

In these two hospitals, high level of ignorance noticed regarding the procedure and indications of basic electrosurgical equipment was seen. There is a need to raise awareness and provide necessary training on how to safely use these devices.

## Ethical Approval

Ethical Approval wasn't required. The study wasn't involving any patients or any interventions. It was a cross sectional survey and no approval was sought.

## Sources of funding

All funding was provided by the authors themselves. No external funding was needed.

## Author contribution

**1. Awais Amjad Malik**: Conceptualization, Methodology, Project Administration.

**2. Romaisa Shamim Khan**: Conceptualization, Methodology, Writing original draft.

**3. Ruqayya Naheed Khan**: Data Curation, Writing original draft.

**4. Osama Shakeel**: Data Curation, Writing original draft.

**5. Hashim Hussnain Ahmed**: Writing Review and editing.

**6. Uzair Rashid**: Methodology, Data curation.

**7. Anam Fatima**: Methodology, Writing Review and editing.

**8. Muhammad Farooq Afzal**: Writing Review and editing, Visualization.

**9. Shahid Khattak:** Visualization, Supervision.

**10. Amir Ali Syed:** Conceptualization, Project Administration.

## Trial registry number

1. Name of the registry: Chinese Clinical Trial Registry
2. Unique Identifying number or registration ID: ChiCTR1900025485
3. Hyperlink to the registration (must be publicly accessible): http://www.chictr.org.cn/showprojen.aspx?proj=42280

## Guarantor

Dr. Awais Amjad Malik.

## Provenance and peer review

Not commissioned, externally peer reviewed.

## Declaration of competing interest

None.

## Appendix A Supplementary data

guidelines guidelines
